# Vitamin C enhances cytotoxicity of CAR-and εTRuC-engineered γδ T cells and modulates Ca^2+^, NFAT, NF-κB and ERK signaling in human γδ T cells

**DOI:** 10.3389/fimmu.2026.1759941

**Published:** 2026-08-28

**Authors:** Michal Zarobkiewicz, Lin Ma, Claudia Juraske, Lea Cierna, Léonce Kouakanou, Sonia Maria Krißmer, Gina J. Fiala, Christian Peters, Wolfgang W. Schamel, Dieter Kabelitz

**Affiliations:** 1 Institute of Medical Immunology, Christian-Albrechts University, University Hospital Schleswig-Holstein, Kiel, Germany; 2 Signalling Research Centres BIOSS and CIBSS, Faculty of Biology, University of Freiburg, Freiburg, Germany; 3 Spemann Graduate School of Biology and Medicine (SGBM), University of Freiburg, Freiburg, Germany; 4 Cyto Kiel, Cytometry Core Facility, Christian-Albrechts University, University Hospital Schleswig-Holstein, Kiel, Germany; 5 Centre for Chronic Immunodeficiency (CCI), Medical Centre Freiburg, Faculty of Medicine, University of Freiburg, Freiburg, Germany

**Keywords:** adoptive cell therapy, CAR, immunotherapy, TRuC, vitamin C, γδ T cells

## Abstract

γδ T cells have raised interest as effector cells in cancer immunotherapy, due to their broad and HLA-independent reactivity towards multiple tumor entities. Despite promising ongoing clinical studies, there is a need to improve the effector function and thereby the efficacy of γδ T cells. In addition to its role as anti-oxidant, Vitamin C is an epigenetic modifier and exerts multiple effects on T-cell differentiation. While Vitamin C enhances *in vitro* proliferation, cytokine production and cytotoxicity of γδ T cells, the associated signaling pathways have so far not been elucidated. Here we demonstrate that Vitamin C enhances the cytotoxic effector function of unmodified as well as anti-CD19 chimeric antigen receptor (CAR) and TCR fusion construct (εTRuC)-transduced zoledronate-expanded γδ T cells. This was associated with increased upregulation of CD25 and IFN-γ secretion. Vitamin C increased calcium influx and nuclear translocation of NFAT and NF-κB in short-term expanded γδ T-cell lines. Although Vitamin C did not alter CD3ζ phosphorylation following stimulation with anti-CD3 antibodies, it reduced phosphorylation of ERK1/2 without affecting p38 MAP kinases. Further analysis showed that Vitamin C did not modulate the expression of selected immune checkpoint molecules on γδ T cells. Our results identify important signaling events where Vitamin C enhances the effector activity of human γδ T cells.

## 1 Introduction

Vitamin C (VC), also known as ascorbic acid, is a pleiotropic vitamin with multiple roles in physiology and pathophysiology. VC is an anti-oxidant which reduces reactive oxygen species (ROS) and thus can protect cells from oxidative stress (1). VC also serves as co-factor for multiple enzymatic reactions. Chronic VC deficiency results in scurvy disease due to improper assembly of the extracellular matrix which depends on the hydroxylation of proline and lysine residues by 2-oxoglutarate-dependent dioxygenases, which require VC as a co-factor (2). Moreover, VC is also an important epigenetic modifier, due to its co-factor activity for ten-eleven-translocation (TET) and Jumonij-C domain containing histone demethylase (JHDM) enzymes which are involved in DNA and histone demethylation, respectively (3–5). Not surprisingly, VC exerts multiple effects on the immune system. VC promotes the differentiation of early T-cell progenitors, drives Th1 cell differentiation, and stabilizes regulatory T-cell (Treg) function through demethylation of specific regions in the *FoxP3* gene (6–8). Recent reviews have extensively summarized the impact of VC on T cell activation and differentiation (9–11). VC has been also studied for potential effects in cancer therapy. It directly kills colorectal cancer cells with mutations in *KRAS* and *BRAF* (12). Furthermore, high dose i.v. application of VC enhances the efficacy of immune checkpoint blockade in various mouse tumor models and increases the recruitment of T cells into the tumor microenvironment (13).

γδ T cells have recently attracted broad interest as potent effector cells in cancer immunotherapy, due to their strong cytotoxic potential and HLA-independent tumor cell recognition allowing for allogeneic application across HLA barriers (14–16). Human Vγ9Vδ2 T cells (which account for the majority of peripheral blood γδ T cells) sense microbial or eukaryotic pyrophosphates (“phosphoantigens”, pAg) through the conformational modulation of transmembrane butyrophilin (BTN) molecules BTN3A1/2 and BTN2A1 (17). Tumor cells frequently overproduce endogenous pAg and can then be sensed and killed by Vγ9Vδ2 T cells (18). We have previously studied the effect of VC on the activation and differentiation of human γδ T cells *in vitro*. We observed that the more stable phospho-modified VC (pVC) increased the proliferative expansion and cytokine production of γδ T cells (19) but also induced *FOXP3* demethylation when γδ T cells were cultured with TGF-β together with pVC (20). Furthermore, γδ T cells from healthy donors expanded in the presence of VC for subsequent adoptive transfer into cancer patients displayed higher cytotoxicity than control cells (21).

Adoptive transfer of αβ T cells expressing a chimeric antigen receptor (CAR) has revolutionized the treatment of hematologic malignancies (22). CARs are fusion proteins that combine antibody-derived tumor antigen recognition domains with intracellular signaling domains of the T cell receptor (TCR) and co-stimulatory receptors, such as CD28 or 4-1BB. This design reprograms T cells to recognize and eliminate tumor cells in an human leukocyte antigen (HLA)-independent manner (23). Although originally developed with αβ T cells this concept has now been expanded to CAR γδ T cells (15, 24, 25). In contrast to CARs, in TCR fusion constructs (TRuCs) the antibody-derived binding domain is directly fused to the TCR, such as to the CD3ε subunit, resulting in εTRuCs (26, 27). The activity of the TCR is regulated by conformational changes that CARs do not possess (28–30). Since εTRuCs use the full TCR machinery, their signaling is closer to the one by the TCR, limiting tonic signaling, exhaustion and severe side effects (27). We have previously shown that εTRuCs can also be used in γδ T cells (31).

Relevant for this study, we noted better survival of γδ T cells transduced with an anti-CD19 εTRuC upon expansion in the presence of pVC (31). However, it is not known whether pVC enhances the effector function of εTRuC-expressing γδ T cells and whether this would also be the case for CAR γδ T cells, an effect recently indicated for CAR αβ T cells (32). Further, little information is so far available as to how VC or pVC affects γδ T-cell activation at the molecular level. In the present study we have therefore investigated how pVC affects signaling pathways in γδ T cells at the level of calcium influx, nuclear translocation of NFAT and NF-κB, and MAP kinase activation.

## 2 Materials and methods

### 2.1 Establishment of short-term γδ T-cell lines

Peripheral blood mononuclear cells (PBMC) were isolated from leukocyte concentrates obtained from healthy adult blood donors by Ficoll-Hypaque density gradient centrifugation. Leukocyte concentrates were kindly provided by the Institute of Transfusion Medicine, UKSH Campus Kiel, and the study was approved by the Ethical Committee of the Kiel University Medical Faculty (code D466/22). Selective activation and expansion of Vγ9Vδ2 T cells was performed as reported previously (33). Briefly, PBMC were activated with 2.5 μM zoledronate (Zol; Novartis, Basel, Switzerland) in the presence of 50 IU/mL IL-2 (Proleukin, Roche). The culture medium was RPMI 1640 supplemented with 100 U/mL penicillin, 100 μg/mL streptomycin and 10% heat-inactivated fetal bovine serum (FBS) (complete medium). IL-2 was supplemented every second day, and cell cultures were split as appropriate. After 14 to 21 days, the proportion of Vγ9Vδ2-positive γδ T cells was between 85 and 98%. Experiments were performed with cell lines of at least 85% purity. In some experiments, γδ T cells were expanded in the additional presence of 50 μg/mL phospho-modified Vitamin C (pVC) (L-ascorbic acid 2-phosphate; Merck Millipore, cat. nr. A8940) which is more stable than regular VC (19). pVC was added to γδ T-cell cultures at different time points as indicated under Results. Based on our previous dose-response analysis (19), the same concentration of pVC (50 μg/mL) was used throughout all experiments.

### 2.2 Lentiviral transduction

Lentiviral production and transduction were performed as described previously (31). Briefly, on day 7 in culture, Zol-expanded γδ T cells were adjusted to 1x10^6^ cells/mL in fresh medium with 50 U/mL IL-2 and plated in a 24-well plate. These γδ T cells were lentivirally transduced with multiplicity of infection (MOI) 5 on day 8 after isolation by addition of the virus and soft resuspension. On day 9, new medium, 50 U/mL IL-2 and 50 μg/ml pVC were added and IL-2 and pVC were added every second day, or cells were kept without pVC but with IL-2 over a culture period of additional 4–5 days. Transduction efficacy of the γδ T cells was analyzed by flow cytometry.

### 2.3 Cytotoxicity assays

The pancreatic adenocarcinoma (PDAC) cell line Panc89 was kindly provided by Dr. Christian Röder, University Kiel, Germany). Killing of Panc89 tumor cells by Zol-expanded γδ T-cell lines (34) was assessed by ^51^Cr release assay as described (35). Target cells were labeled with 150 μC sodium ^51^chromate. After washing steps, 2000 target cells were co-cultured for 4 h in 96 well V-shaped microtiter plates with titrated numbers of effector cells in the presence or absence of 300 nM bromohydrin pyrophosphate (BrHPP; Innate Pharma, Marseille, France) with or without 50 μg/mL pVC. In some experiments, Panc89 cells were pretreated for 18 h with 50 μg/mL before use as target cells. Radioactivity in supernatants was measured in a β-counter. Maximum release was determined in Triton-X 100. Specific cytotoxicity was calculated as experimental release – spontaneous release/maximal release – spontaneous release. Spontaneous release did not exceed 15%. In some experiments, Panc-89 cells were pre-treated with pVC and used as target cells.

### 2.4 Luciferase activity-based cell lysis assays

Killing of CD19-expressing Burkitt lymphoma Ramos and B-cell ALL Nalm6 target cells (gift from Dr. Michael Reth, University Freiburg, Germany) by Zol-expanded γδ T cells transduced or not with an anti-CD19 CAR, εTRuC was performed in a luciferase-based cell lysis assay exactly as described before (31). In brief, non-transduced or transduced Vγ9Vδ2 T cells, untreated or pre-treated with pVC were used as effector cells and co-cultured with the luciferase-expressing target cells (Nalm6 and Ramos) at an effector/target E/T ratio of 1:1. A total of 200.000 cells (sum of the effector and target cells) were seeded in triplicates in a 96-well flat-bottom plates in complete medium with IL-2. Addition of 75 μg/mL D-luciferin allowed to measure the relative luminescence (RLU) using a Tecan Infinite M200 Pro plate reader. Maximum death RLU was determined in samples by adding Triton X-100, while spontaneous death was determined by culturing the target cells alone. RLU was measured after 8 h of co-culture and specific lysis was then calculated as follows: % specific lysis = 100 × [(spont. death RLU - test RLU)/(spont. death RLU - max. death RLU)].

### 2.5 Quantification of cytokine secretion

Supernatants of co-cultures were collected after 24 h to determine the concentration of released tumor necrosis factor α (TNF-α) and interferon γ (IFN-γ) by the non-transduced or transduced Vγ9Vδ2 T cells, untreated or pre-treated with pVC. Human TNF-α and IFN-γ ELISA kits were obtained from Invitrogen (TNF-α, lot 396302-010, analytical sensitivity 4 pg/mL; IFN-γ, lot 423448-002, analytical sensitivity 8 pg/ml).

### 2.6 Flow cytometric analysis of Vγ9Vδ2 T-cell viability, purity and CD8 expression

γδ T cells were washed with PBS/2% FBS and stained with a viability dye together with BV421 labeled CD3 (Biolegend), PE-labeled γδ TCR (Invitrogen), Alexa Fluor 647-labeled anti-CD8α (BioLegend) antibodies. Samples were acquired using an Attune NxT flow cytometer and analyzed using FlowJo 10 software. γδ T cell viability was determined by the forward scatter and side scatter. Purity was defined as the percentage of CD3-positive, γδ TCR-positive cells among live singlet lymphocytes. CD4 and CD8 expression was analyzed within the live CD3-positive, γδ TCR-positive population.

### 2.7 Flow cytometric analysis of CAR and εTRuC transduction efficiency

CAR- or εTRuC-transduced γδ T cells were washed with PBS/2% FBS and stained with a viability dye, Alexa Fluor 488-labeled anti-CD3 (BioLegend), PE-labeled anti-γδ TCR (Invitrogen), and APC-labeled anti-FLAG or APC-labeled anti-mouse F(ab’_2_) antibodies (for the εTRuC). Samples were acquired using an Attune NxT flow cytometer and analyzed using FlowJo 10 software. CAR or εTRuC transduction efficiency was determined as the percentage of FLAG or F(ab’_2_) and BFP positive cells within the live CD3-positive, γδ TCR-positive lymphocyte population.

### 2.8 Analysis of CD25, CD4, and CD8 expression following co-culture

Cells of the tumor cell, γδ T-cell co-cultures were washed after 24 h with PBS/2% FBS and stained with APC-labeled anti-CD25 (eBioscience), BV421-labeled anti-CD19 (BioLegend) and FITC-labeled anti-γδ TCR (Life Technologies), PE-labeled anti-CD8α (Beckman Coulter), and PE-Cy7-labeled anti-CD4 (BioLegend) antibodies. Cells were measured by an Attune NxT flow cytometer and analyzed using FlowJo 10 software. CD25 expression was analyzed with a gate on CD19-negative, γδ TCR-positive, live lymphocytes. The distribution of CD4 and CD8 expression was further analyzed within the CD25 positive γδ T cell population.

### 2.9 CD107a degranulation assay

Tumor cells and γδ T cells were co-cultured in the presence of PE-labeled anti-CD107a antibody. After 6 h, cells were collected, washed with PBS/2% FBS, and stained with a viability dye, FITC-labeled anti-γδ TCR (Life Technologies), and BV421-labeled anti-CD19 (BioLegend) antibodies. Samples were acquired using an Attune NxT flow cytometer and analyzed using FlowJo 10 software. CD107a expression was determined within the CD19-negative, γδ TCR positive live singlet lymphocyte population.

### 2.10 Intracellular staining of granzyme B and perforin

Unstimulated γδ T cells were incubated with brefeldin A for 3 h. Cells were collected, washed with PBS/2% FBS Cells and subsequently stained with a viability dye, BV421-labeled anti-CD3, and FITC-labeled anti-γδ TCR antibodies. After surface staining, the cells were fixed and permeabilized and intracellularly stained with APC-labeled anti-granzyme B and PE-conjugated anti-perforin antibodies (BioLegend). Samples were acquired using an Attune NxT flow cytometer and analyzed using FlowJo 10 software. Intracellular granzyme B and perforin expression was determined within the live CD3-positive, γδ TCR-positive singlet lymphocyte population.

### 2.11 Measurement of Ca^2+^ influx

Zol-expanded γδ T-cell lines were preincubated or not for 4 or 24 h with 50 μg/mL pVC. Calcium flux was assessed using Fluo-3 AM and Fura Red AM dyes. Cells were resuspended at 5×10^6^ cells/mL in a staining solution composed of 1.3 μL Fluo-3 AM (1 mM stock; Life Technologies), 2.75 μL Fura Red AM (1 mM stock; Invitrogen), and 5 μL Pluronic F-127 (20% stock; Invitrogen) in 1mL RPMI 1640 (1% FCS). Following incubation, cells were washed and resuspended in fresh RPMI 1640 (1% FCS) at 1×10^7^ cells/mL. Immediately before acquisition, cells were diluted in prewarmed RPMI 1640 (1% FCS) to a final concentration of 0.5×10^6^ cells/mL. Calcium mobilization was measured using a BD FACS Canto with a flow rate of 500–1000 events/sec. Baseline fluorescence was recorded for 60 seconds, after which cells were stimulated with 1 μg/mL anti-CD3 (UCHT1; Biolegend) with or without pVC. Measurement continued for an additional 6 minutes. Data were analyzed using FlowJo. A calcium flux parameter was calculated as: Calcium = (Fluo-3/Fura Red) × 100. Baseline was defined as the initial 60 s.

### 2.12 Nuclear translocation of NFAT and NF-κB

γδ T cells were expanded with zoledronate in the absence [Medium] or presence of pVC [pVC line]. After the expansion, γδ T cells were pre-treated with pVC for 24 h [pVC 24h pre] or were left untreated. The γδ T cells were then re-stimulated for 4 h with anti-CD3 (UCHT1) 1 µg/mL. To some of the untreated γδ T cells, pVC was added during this re-stimulation [pVC assay]. A, B) The translocation of NFAT (αNFAT, clone 7A6; yellow) and NF-κB (anti-NF-κB, clone 14G10A21; blue) into the nucleus (syto deep red; red) was analyzed using ImageStream *X Mark II* (Merck) flow cytometric microscopy. The integrated software INSPIRE (version 200.1.388.0, Merck) was used for data collection of raw image files.

### 2.13 Phosflow and Western Blot for activation of kinases

Zol-expanded γδ T-cell lines pre-treated or not with pVC were stimulated in prewarmed medium with 2 μg/mL anti-CD3 mAb UCHT1 with incubation times of 1, 3, 5, 10, and 30 min. Reactions were stopped by adding 200 μL cold PBS, followed by immediate centrifugation. Cells were fixed in 1% PFA, washed twice with PBS, and permeabilized with ice-cold methanol at 4 °C for at least 30 min. Samples were stained with phospho-specific antibodies: Alexa Fluor 647-CD3ζ (BD, cat. Nr. 558489), Alexa Fluor 647-p38 (BD, cat. Nr. 562066), Alexa Fluor 647-Akt (BD, cat. Nr. 561670), and PE-ERK1/2 (BD, cat. Nr. 612566). Samples were acquired with BD FACS Canto. In addition, phosphorylation of ERK1/2 and p38 was also detected by Western blot. 1.5×10^6^ Zol-expanded γδ T cells were activated for 1 to 60 min with 4 μg/mL UCHT1. Cell pellets were lysed in NP-40 lysis buffer with protease inhibitors and phosphatase inhibitor cocktail (Phosphatase Inhibitor Cocktail IV, Thermo Fisher). Proteins were separated by SDS-PAGE using 4–12% Bis-Tris gels (ThermoFisher, cat. Nr. NP0321BOX). Precision Plus All Blue ladder (Bio-Rad, cat. Nr. 1610373) was loaded alongside samples. Proteins were transferred to 0.45 μm membranes. Membranes were blocked with 5% BSA for 1 h at room temperature. Membranes were incubated overnight at 4 °C with primary antibodies in TBS-T. Following three TBS-T washes, membranes were incubated with HRP-conjugated secondary antibodies for 45 min at room temperature and washed again. Chemiluminescent detection was performed using ECL substrate, applied for 1 min. The following antibodies were used for WB analysis: beta-actin (Sigma, cat. Nr. A2228), beta-actin HRP (Proteintech, cat. Nr. HRP-60008), p-p38 (Cell Signaling Technology, cat. Nr. 9211S), p-ERK1/2 (Cell Signaling Technology, cat. Nr. 9101S). Semiquantitative analysis of ERK1/2 phosphorylation was done by densitometry.

### 2.14 Expression of checkpoint molecules on Zol lines

Zol-expanded γδ T-cell lines were incubated for 1 and 24 h in the absence or presence of 50 μg/mL pVC and 300 nM BrHPP. The surface expression of checkpoint molecules was detected with the following antibodies: PD-1 PE (clone: EH12.1, cat. Nr. 560795, BD), TIM-3 PE (clone: 7D3, cat. Nr. 563422, BD), TIGIT PE (clone: TgMab-2, BD, cat. Nr. 568672). All samples were measured using a FACS Canto I and analyzed with the BD FACSDiva software v.8. The FlowJo software v.10 was used for further analysis.

### 2.15 Statistical analysis

Data are represented as mean ± SD. All differences between experimental groups were analyzed with the Student´s t-test or 2-way ANOVA using GraphPad Prism (v8.4.3; GraphPad Software), or Microsoft Excel (2024). Significant differences were assumed for p values smaller than 0.05. (ns = non-significant, *p < 0.05, **p < 0.01, ***p < 0.001, and ****p < 0.0001). n refers to the number of independently performed experiments.

## 3 Results

### 3.1 pVC enhances cytotoxicity and IFN-γ secretion in γδ T cells

**Figure 1:**
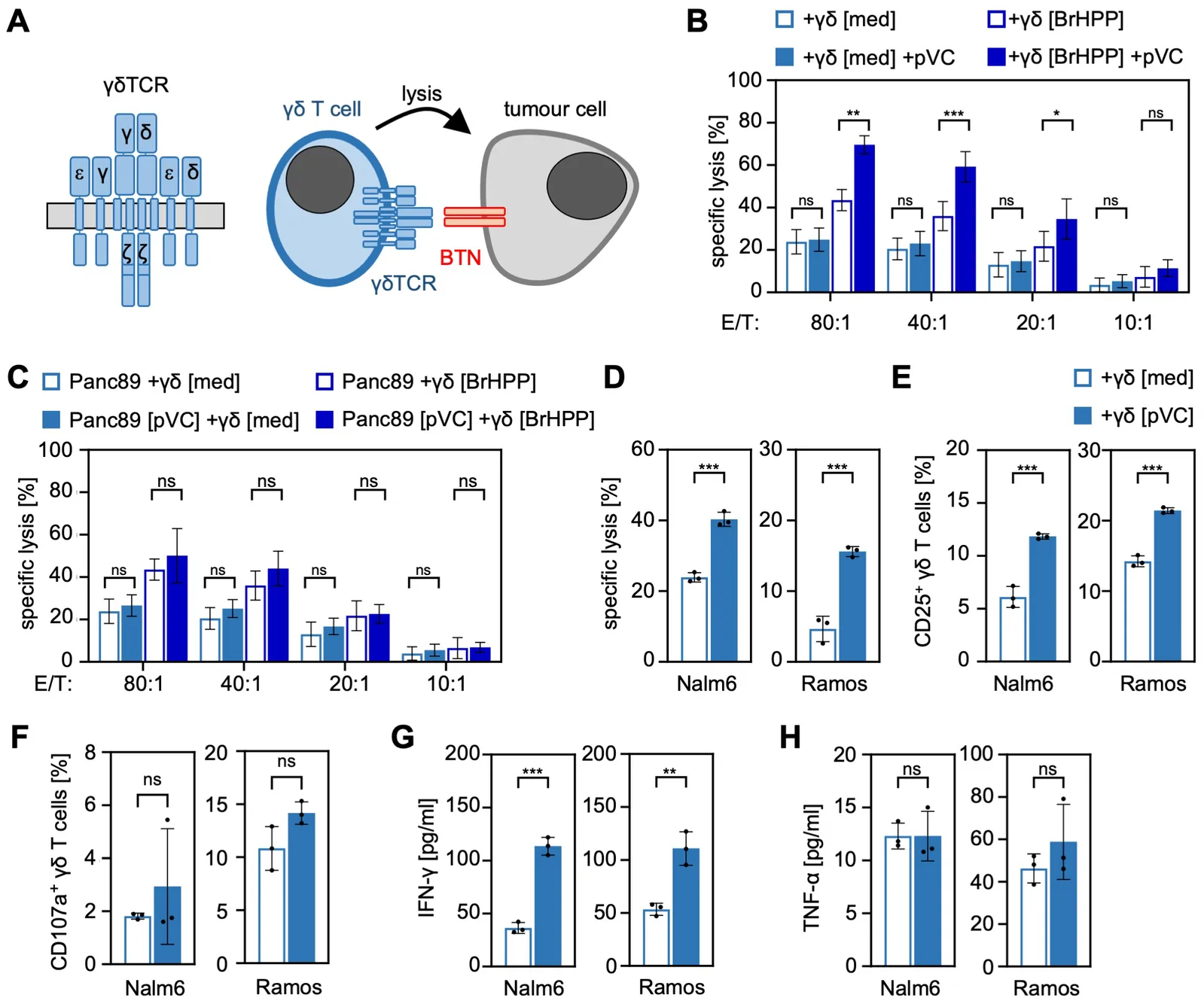
pVC enhances tumor cell lysis by Vγ9Vδ2 T cells. **(A)** Scheme of the γδ TCR and lysis of tumor cells by Vγ9Vδ2 T cells. **(B)** Panc89 cells were ^51^Cr labelled and co-cultured with Zol-expanded Vγ9Vδ2 T cells at different effector/target (E/T) ratios. T cells were left unstimulated (γδ[med]) or re-stimulated with BrHPP (γδ[+BrHPP]) and pVC was added to the co-culture or not. The bar diagram shows the quantification of the released ^51^Cr as percentage of specific lysis. Significance was analyzed by 2way ANOVA (n=4). **(C)** Untreated Panc89 and Panc89 cells pretreated with pVC for 18 h [pVC]) were ^51^Cr labelled and co-cultured with unstimulated or BrHPP-re-stimulated Vγ9Vδ2 T cells. Measurement and analysis as in **(B)**. Significance was analyzed by 2way ANOVA (n=4). **(D-G)** Nalm6 and Ramos cells expressing luciferase were co-cultured with Zol-expanded Vγ9Vδ2 T cells untreated (γδ[med]) or pre-treated with pVC (γδ[pVC]) at a ratio of 1:1. **(D)** After 8 h, luciferase activity was recorded and percentage of specific lysis was calculated. Significance was analyzed by unpaired t test (n>3; shown one representative experiment in triplicates). **(E)** After 24 h, cells of the co-culture were stained for CD25, CD19 and pan-TCR and analyzed by flow cytometry. A bar diagram of the percentage of CD25-positive γδ T cells is shown. Significance was analyzed by unpaired T test (n=3; shown one representative experiment in triplicates). **(F)** After 6 h of co-culture in the presence of an anti-CD107a antibody, cells were stained for CD19, γδ TCR, and viability and analyzed by flow cytometry. Bar graphs show the percentage of CD107a-positive γδ T cells. Significance was analyzed by unpaired T test (n=3; one representative experiment in triplicates is shown). **(G, H)** After 24 h of co-culture the supernatants were used to determine IFNγ and TNF-α. The significance was calculated by unpaired T test (n=3; shown one representative experiment in triplicates). *p<0.05, **p<0.01, ***p<0.001, ns, not significant.

γδ T cells expanded in the presence of VC have been previously reported to exert increased cytotoxic function (21). In addition to unmodified γδ T cells such as Zol-expanded Vγ9Vδ2 and Vδ1 “Delta One T” (DOT) cells, γδ T cells expressing transduced CAR constructs are also in clinical development (15, 16). Therefore, we reanalyzed the effect of pVC on the killing capacity of γδ T cells in several *in vitro* systems. In all experiments we used the phospho-modified L-ascorbic acid 2-phosphate (pVC) which is more stable than the unmodified VC and does not acidify the culture medium (19). In a first setting we analyzed unmodified γδ T cells co-cultured with Panc89 PDAC or Ramos or Nalm6 B-cell lymphoma cells (see schematic graph in **Figure 1A**). As shown in **Figure 1B**, Zol-expanded γδ T cells exerted limited killing of pancreatic adenocarcinoma Panc89 target cells at various E/T ratios, even in the presence of pVC. While TCR activation by phosphoantigen BrHPP increased the killing, the additional presence of pVC further boosted tumor lysis at all tested E/T ratios, indicating that pVC enhances TCR signaling pathways leading to enhanced cytotoxic activity. Of note, pre-incubation of Panc89 tumor cells for 18 h with pVC did not modulate their sensitivity towards γδ T cells (**Figure 1C**). This indicates that the enhanced tumor cell lysis in **Figure 1B** was due to an enhanced activity by the γδ T cells. Likewise, pre-incubation of Zol-expanded γδ T cells with pVC significantly enhanced killing of the B-cell lymphomas Nalm6 and Ramos (**Figure 1D**), demonstrating that the boosting effect was not limited to killing of the pancreatic tumor cell line.

Increased tumor lysis by pVC-treated γδ T cells could be due to enhanced T-cell activation. Indeed, when the Zol-expanded γδ T cells were co-cultured with the B-cell lymphoma lines, the pre-treatment with pVC induced higher levels of activation as evidenced by CD25 up-regulation (**Figure 1E**). Our gating strategy to identify γδ T cells in flow cytometry is shown in **Supplementary Figure 1A** and their purity was around 90% (**Supplementary Figure 1B**).

A subpopulation of γδ T cells expresses the CD8 co-receptor. These CD8^+^ γδ T cells produce cytotoxic mediators and are differentially affected by pathological settings (36–38). Hence, we tested whether pVC treatment had an impact on the abundance of CD8^+^ γδ T cells within the activated cells in Nalm6 and Ramos co-cultures. Staining for CD8 revealed that only a minor fraction of the activated (CD25-positive) γδ T cells expressed CD8 (**Supplementary Figure 1C**). pVC treatment did not change the percentage of CD8^+^ cells among CD25^+^ γδ T cells during the co-cultures (**Supplementary Figure 1C**). In fact, the percentage of CD8^+^ γδ T cells in our Zol cultures is in the same range (3-5%, data not shown). Therefore, γδ T activation by the tumor cells did not change this ratio, indicating that pVC treatment did not preferentially sensitize CD8^-^ or CD8^+^ γδ T cells for activation.

Cytotoxicity can be mediated by degranulation, secretion of IFN-γ or TNF-α among other mechanisms. To obtain insight, we evaluated degranulation by measuring CD107a on the surface of the γδ T cells after co-culture with Nalm6 and Ramos. Although differences in CD107a surface expression did not reach statistical significance, Zol-expanded γδ T cells pre-incubated with pVC tended to display higher CD107a levels upon co-culture with Ramos cells (**Figure 1F**), suggesting enhanced degranulation. The amounts of granzyme B and perforin in the γδ T cells was the same with or without pVC (**Supplementary Figure 1D**), indicating that the content of the granula might be similar. In addition, pre-incubation with pVC increased IFN-γ secretion (**Figure 1G**), while levels of secreted TNF-α were not different (**Figure 1H**). In conclusion, pVC treatment might enhance tumor killing by promoting IFN-γ production.

### 3.2 In engineered γδ T cells pVC enhances cytotoxicity and IFN-γ secretion upon stimulation via their CAR or εTRuC

**Figure 2:**
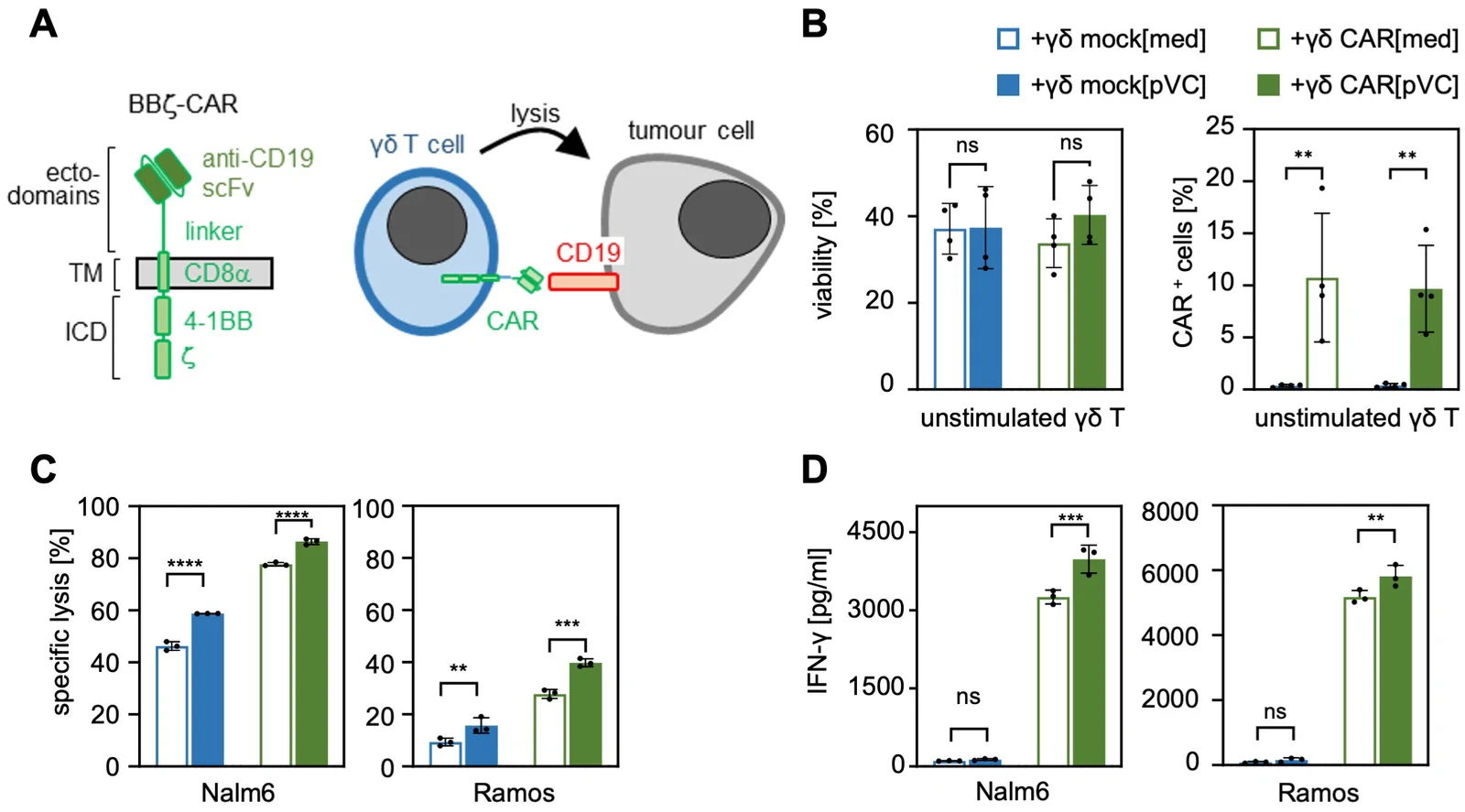
pVC enhances tumor cell lysis by anti-CD19 CAR-expressing Vγ9Vδ2 T cells. **(A)** Scheme of the anti-CD19 BBζ-CAR and lysis of tumor cells by anti-CD19 CAR-expressing Vγ9Vδ2 T cells. **(B)** After 14 days of expansion, the viability and transduction efficiency of mock- or CAR-transduced Vγ9Vδ2 T cells, pre-treated or not with pVC, were analyzed by flow cytometry. Bar diagrams show the percentages of viable cells (as determined from the forward scatter - side scatter plot) and CAR-positive cells. CAR expression was analyzed within live γδ T cells. Significance was analyzed by paired T test (n =4). **(C, D)** Luciferase-expressing Nalm6 and Ramos cells were co-cultured with Zol-expanded Vγ9Vδ2 T cells which had been transduced with mock (γδ mock) or anti-CD19 BBζ-CAR (γδ CAR) lentiviral vectors and pre-treated or not with pVC at a ratio of 1:1. **(C)** After 8 h, luciferase activity was recorded and percentage of specific lysis was calculated. Significance was analyzed by 2-way ANOVA (n=4; one donor in triplicates is shown). **(D)** After 24 h, the supernatants of the co-cultures were used to determine IFN-γ. The significance was calculated by 2-way ANOVA (both n=4; one donor in triplicates is shown). **p<0.01, ***p<0.001, ****p<0.0001, ns, not significant.

γδ T cells can be reprogrammed to recognize CD19-positive tumor cells by expressing anti-CD19 CARs (25, 39, 40). Here, we asked if pVC can also augment the killing activity of anti-CD19 CAR-transduced γδ T cells. We used the FDA-approved CAR containing the cytoplasmic tails of 4-1BB and CD3ζ (**Figure 2A**). A schematic graph of the interaction of anti-CD19 CAR-expressing γδ T cells with CD19-positive target cells is shown in **Figure 2A**. Our gating strategy to identify CAR-transduced γδ T cells in flow cytometry is shown in **Supplementary Figure 2A**. Again, the purity of the Vγ9Vδ2 T cells was around 90% (**Supplementary Figure 2B**). Flow cytometric analysis showed that CAR γδ T cells cultured with pVC showed the same viability as cells in medium alone (**Figure 2B**). As expected, anti-CD19 BBζ-CAR-transduced γδ T cells expressed the CAR (**Figure 2B** and **Supplementary Figure 2A**) and pVC treatment did not change the percent of transduced Vγ9Vδ2 T cells. However, pre-incubation with pVC significantly enhanced the specific lysis of both Nalm6 and Ramos cells by BBζ-CAR-expressing γδ T cells as well as lower level non-CAR-mediated killing induced by mock-transduced γδ T cells (**Figure 2C**). The same result was obtained in two independent donors, while the other two donors showed a similar trend that did not reach statistical significance (**Supplementary Figure 2C**). Additionally, pre-incubation with pVC increased IFN-γ secretion (**Figure 2D**). This result was statistically significant in two independent donors, while the other donor did not reach statistical significance (not shown).

**Figure 3:**
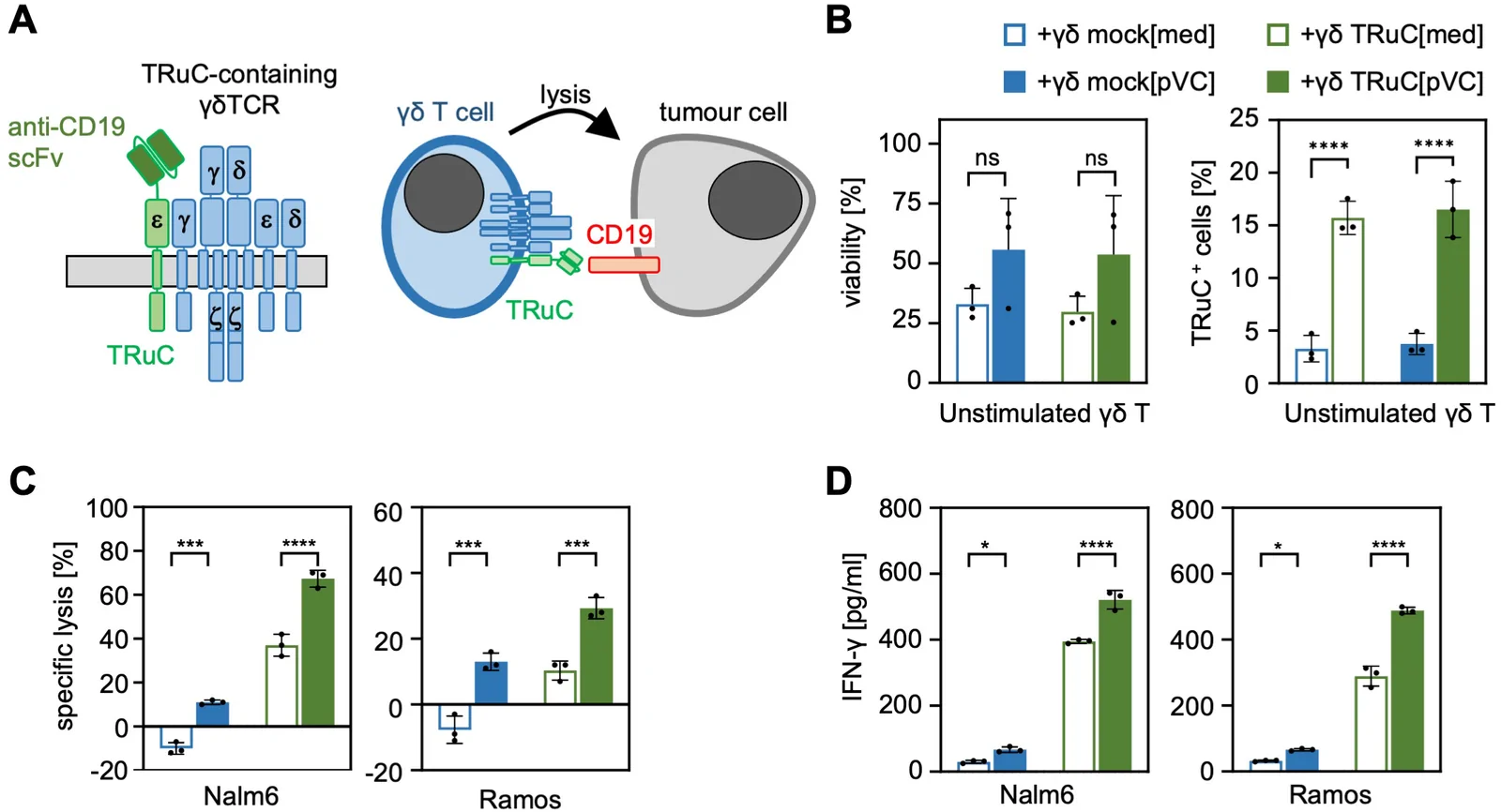
pVC enhances tumor cell lysis by anti-CD19 εTRuC-expressing Vγ9Vδ2 T cells. **(A)** Scheme of the anti-CD19 εTRuC-containing γδ TCR and lysis of tumor cells by anti-CD19 εTRuC-expressing Vγ9Vδ2 T cells. **(B)** After 14 days of expansion, the viability and transduction efficiency of mock- or anti-CD19 εTRuC-transduced Vγ9Vδ2 T cells, pre-treated or not with pVC, were analyzed by flow cytometry. Bar diagrams show the percentages of viable cells and εTRuC-positive cells. CAR expression was analyzed within live γδ T cells. Significance was analyzed by paired T test (n =3). **(C, D)** Luciferase-expressing Nalm6 and Ramos cells were co-cultured with Zol-expanded Vγ9Vδ2 T cells which had been transduced with mock (γδ mock) or anti-CD19 εTRuC (γδ εTRuC) lentiviral vectors and pre-treated or not with pVC at a ratio of 1:1. **(C)** After 8 h, luciferase activity was recorded and percentage of specific lysis was calculated. Significance was analyzed by 2-way ANOVA (n=3; one donor in triplicates is shown). **(D)** After 24 h, the supernatants of the co-cultures were used to determine IFN-γ. The significance was calculated by 2-way ANOVA (both n=3; one donor in triplicates is shown). *p<0.05, ***p<0.001, ****p<0.0001, ns, not significant.

We have recently shown that γδ T cells can also be reprogrammed to recognize CD19-positive tumor cells by expressing an anti-CD19 ϵTRuC (31). In contrast to CARs, the ϵTRuC integrates into the TCR complex (**Figure 3A**), thus making use of the full signaling potential of the TCR (27). To determine whether pVC also enhanced the activity of an ϵTRuC, we generated Zol-expanded Vγ9Vδ2 T cells expressing an anti-CD19 ϵTRuC (**Figure 3A**). The gating strategy to identify ϵTRuC-expressing γδ T cells is shown in **Supplementary Figure 3A**. The purity of the Vγ9Vδ2 T cells was around 90% (**Supplementary Figure 3B**). pVC did not significantly affect viability, although a trend toward increased viability was observed (**Figure 3B**). Flow-cytometric analysis confirmed consistent ϵTRuC expression independent of the presence of pVC, demonstrating the successful generation of anti-CD19 ϵTRuC γδ T cells (**Figure 3B**). pVC significantly enhanced tumor-cell killing in two of three independent donors (**Figure 3C**), while the remaining donor showed a similar but non-significant trend (**Supplementary Figure 3C**). Increased IFN-γ secretion upon pVC treatment was observed in two of three donors (**Figure 3D**), whereas TNF-α secretion was not significantly altered by pVC (**Supplementary Figure 3D**). Both finding are in line with those on unmodified Vγ9Vδ2 T cells (**Figures 1G, H**).

Taken together, we conclude that pVC increases the tumor killing, activation and IFN-γ secretion of γδ T cells as determined independently in several experimental systems although some donor to donor variability was observed. This held true for genetically modified and unmodified γδ T cells.

### 3.3 pVC increases calcium influx in γδ T cells

**Figure 4:**
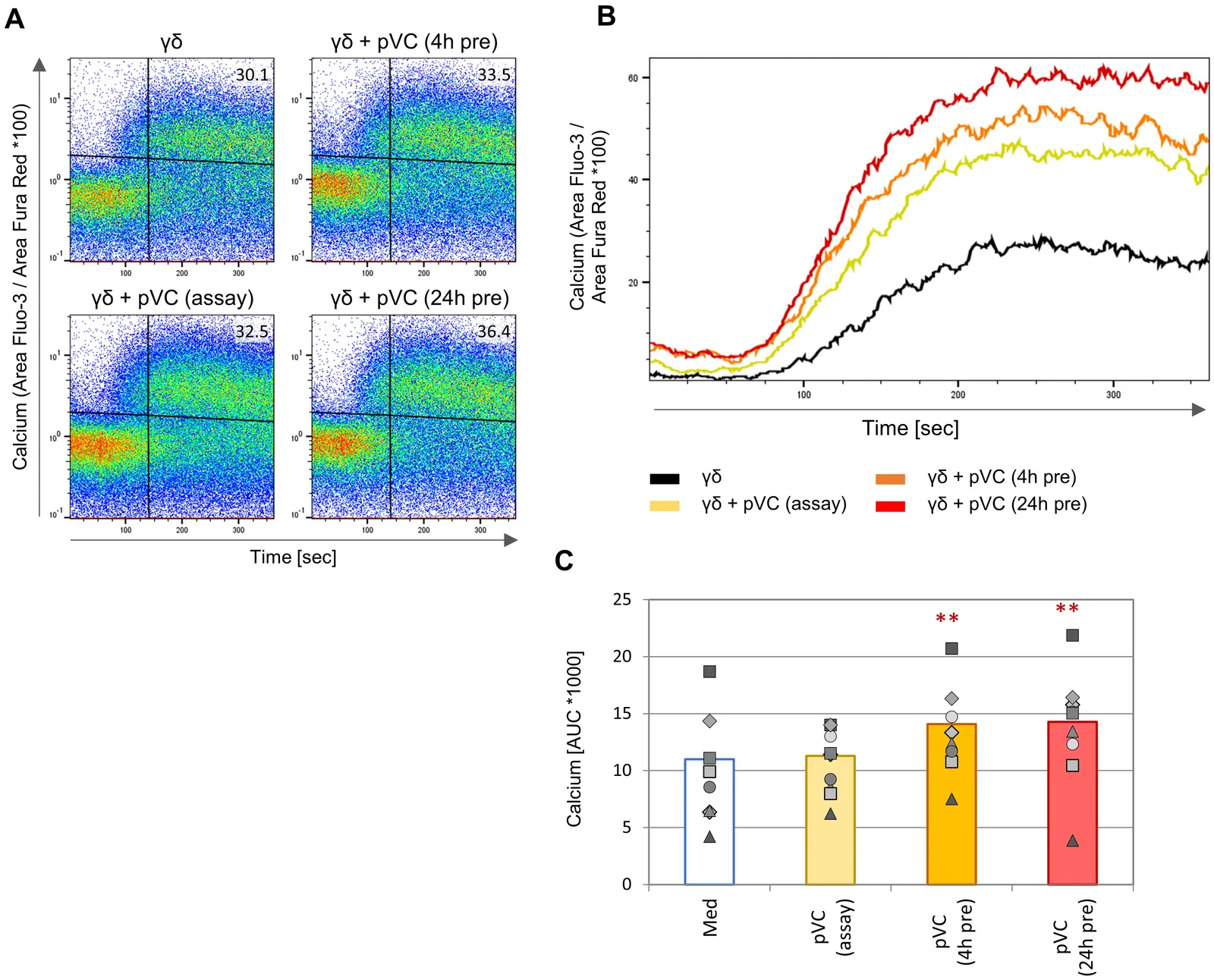
pVC-dependent modulation of Calcium influx in Vγ9Vδ2 T cells. Vγ9Vδ2 γδ T cells were expanded with Zol for 21 days. After expansion, γδ T cells were left untreated [Med], were pre-treated with 50 µg/mL pVC for 4 h [pVC (4 h pre)], or 24 h [pVC (24 h pre)], or pVC was added directly to the assay [pVC (assay)]. The calcium flux was measured using Fluo-3 AM and Fura Red AM dyes. The baseline fluorescence was measured for the first 60 s followed by anti-CD3 (1µg/mL) stimulation and continued measurement for additional 6 min. The calcium flux parameter [Calcium] was calculated as the quotient of Fluo-3/Fura Red and is depicted as percent above baseline. **(A)** Dot plots and **(B)** the kinetics of the derived calcium parameter from a representative experiment. **(C)** The area under the curve [AUC] for the calcium parameter is depicted for 9 independent experiments. Columns represent mean values; asterisks indicate significant difference relative to untreated cells [Med] according to Student´s t-test for paired data. *p ≤ 0.05, **p ≤ 0,01.

The above results indicated that pVC enhances the activation of γδ T cells at the level of CD25 upregulation, IFN-γ secretion and cytotoxicity. TCR-dependent (and thus also εTRuC-containing TCR-dependent) T-cell activation is associated with the rapid mobilization and influx of extracellular calcium. Therefore, we addressed the effect of pVC on the calcium influx upon activation of Zol-expanded γδ T-cell lines with anti-CD3 mAb UCHT1. To this end, γδ T cells were pre-incubated for 4 or 24 h with pVC; alternatively, pVC was added directly to the assay. γδ T cells were activated with anti-CD3 antibody UCHT1, and calcium influx was recorded for several minutes. Representative dot plots are shown in **Figure 4A**. and the calcium influx over time (5 min) under various conditions of pVC treatment of the same cells in **Figure 4B**. A summary of several experiments is depicted as calculated AUC in **Figure 4C**. As can be seen, Zol-expanded γδ T-cell lines preincubated for 4 or 24h with pVC showed a significantly enhanced calcium mobilization when compared to the untreated controls, while direct addition of pVC together with UCHT1 had variable effects. Therefore, increased calcium influx is an early molecular event associated with the stimulatory effect of pVC on γδ T cells.

### 3.4 pVC enhances nuclear translocation of NFAT and NF-κB

**Figure 5:**
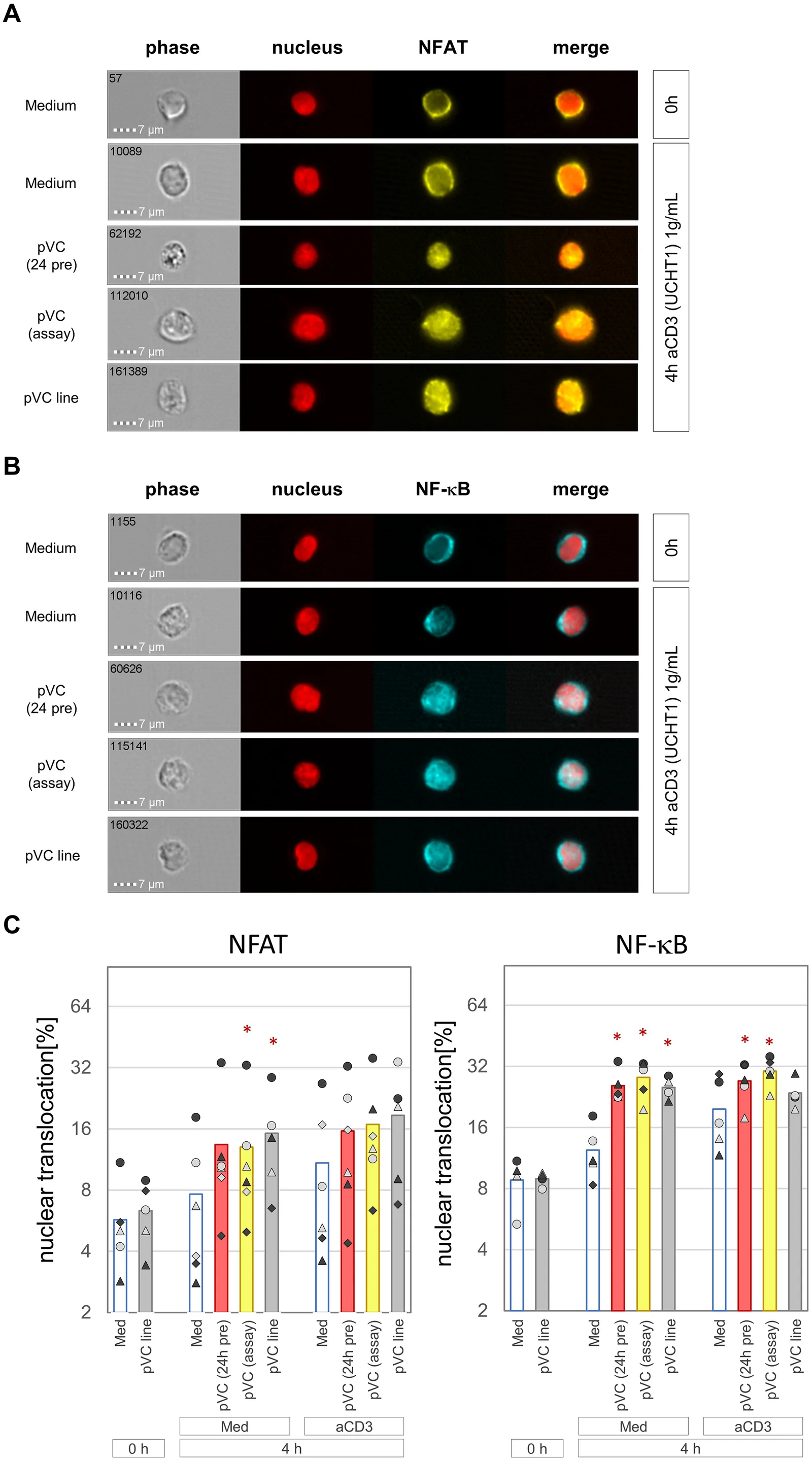
Effect of pVC on nuclear translocation of NFAT and NF-κB in Vγ9Vδ2 T cells. Vγ9Vδ2 γδ T cells were expanded with Zol in the absence [Medium] or presence of pVC [pVC line]. After the expansion, γδ T cells were pre-treated or not with pVC for 24 h [pVC (24h pre)]. The γδ T cells were then re-stimulated for 4 h with anti-CD3 antibody UCHT1. To some of the pVC untreated γδ T cells, pVC was added during this re-stimulation [pVC assay]. **(A, B)** The translocation of NFAT (yellow) and NF-κB (blue) into the nucleus (red) was analyzed using Image Stream flow cytometric microscopy. A representative picture of the nuclear translocation of NFAT **(A)** or NF-κB **(B)** after 0 or 4 h is shown for all different pVC treatments [Medium, pVC (24h pre), pVC assay, pVC line]. **(C)** The percentages of nuclear translocation after 0 and 4 h are shown for NFAT (n=6) and NF-κB (n=5). Columns represent mean values; asterisks indicate significant difference relative to untreated cells [Med] according to Student´s t-test for paired data. *p ≤ 0.05, **p ≤ 0.01.

Nuclear translocation of the transcription factors NFAT and NF-κB is a rapid event following TCR activation and down-stream of calcium influx. Zol-expanded γδ T-cell lines were preincubated for 24 h with pVC [pVC (24h pre)] or not (medium), or pVC was added directly during activation [pVC assay]. We also included Zol-expanded γδ T-cell lines generated in the continuous presence of pVC [pVC line]. Cells were activated or not for 4 h with anti-CD3 antibody UCHT1 and processed for Imagestream cytometry. Representative images of nuclear translocation of NFAT and NF-κB are shown in **Figures 5A, B**, respectively, and a summary of five experiments is presented in **Figure 5C**. CD3 activation alone induced minor translocation of NFAT and NF-κB which was moderately increased in all conditions where pVC was present. Interestingly, the presence of pVC alone without anti-CD3 stimulation already increased nuclear translocation of NFAT and NF-κB (**Figure 5C**). For comparison, we also analyzed cells which had been stimulated for 24 h with anti-CD3 antibody UCHT1 (**Supplementary Figure 4**).

### 3.5 MAP kinase activation

**Figure 6:**
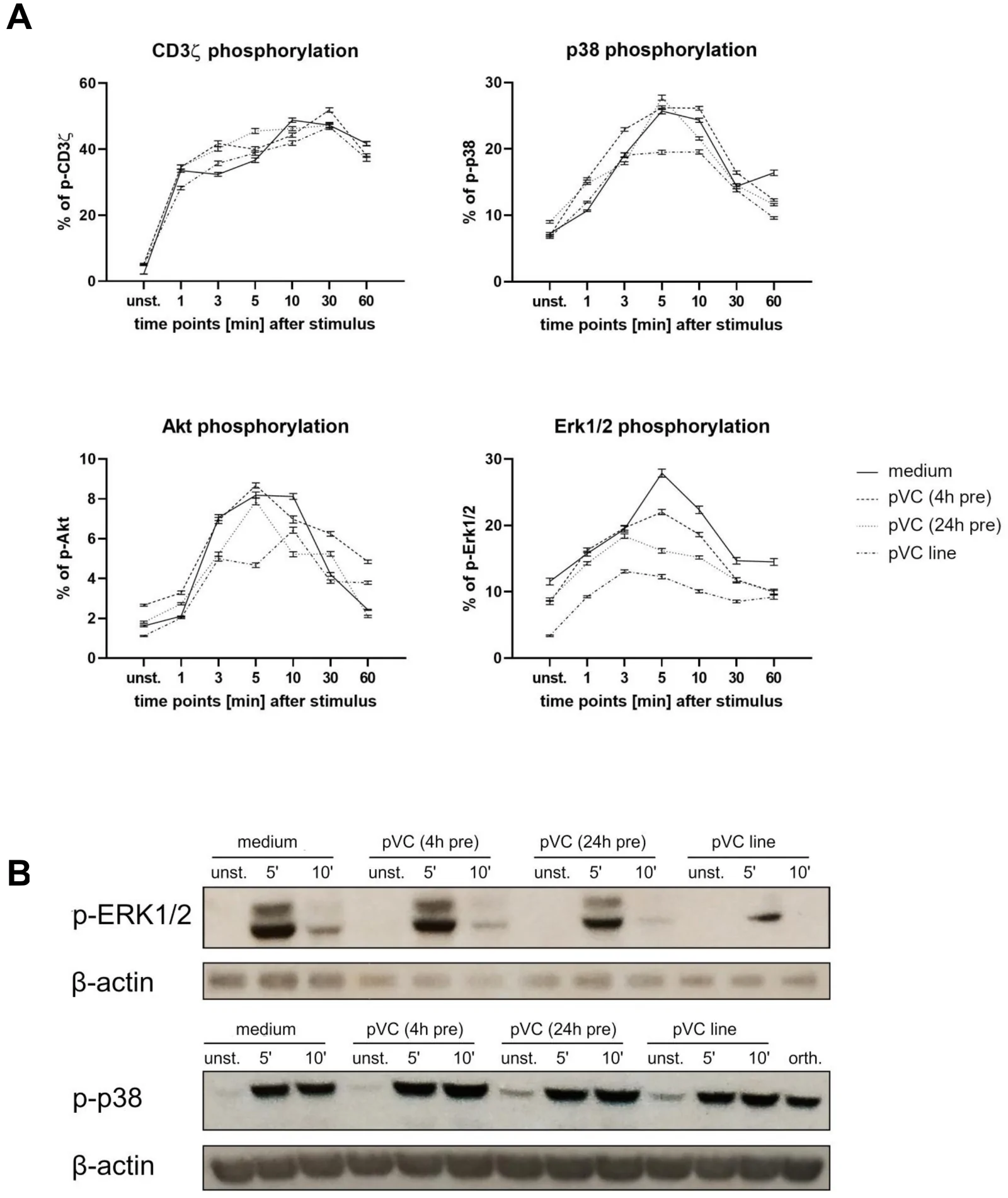
Effect of pVC on MAP kinase activation in Zol-expanded γδ T cells. **(A)** Zol-expanded γδ T cells were pre-incubated or not for 4 h [pVC (4h pre)] or 24 h [pVC (24h pre)] with pVC, or were expanded in the presence of pVC [pVC line]. γδ T cells were activated with 5 μg/mL anti-CD3 mAb UCHT1 and analyzed by Phosflow for the expression of the indicated phosphoproteins over a time period of 60 min. Mean values of three independent experiments are shown. **(B)** γδ T cells as in **(A)** were kept unstimulated or were stimulated for 5 and 10 min with anti-CD3 mAb UCHT1. Subsequently, the expression of p-ERK1/2 (upper panel) and p-p38 (lower panel) was analyzed by Western blotting. β-actin was used as a control. One representative out of three experiments is shown.

Next, we analyzed possible effects of pVC on the rapid phosphorylation of key signaling molecules including CD3ζ, Akt and MAP kinases p38 and ERK1/2 by flow cytometry and Western blot (**Figure 6**). Zol-expanded γδ T cells were pre-incubated (or not) for 4 h [pVC (4h pre)] or 24 h [pVC (24h pre)] with pVC, or were expanded in the presence of pVC [pVC line]. Upon activation with anti-CD3 mAb UCHT1, phosphorylation was measured by Phosflow (**Figure 6A**). While pVC did not modulate the intensity and kinetics of CD3ζ phosphorylation (**Figure 6A**, upper left), γδ T cells pre-treated with pVC (particularly those expanded in the presence of pVC) displayed reduced ERK1/2 phosphorylation as evidenced by Phosflow (**Figure 6A**, lower right) and confirmed by Western blot (**Figure 6B**, upper panel). The ERK1/2 phosphorylation of three independent experiments was quantified by densitometry (**Supplementary Figure 5**). γδ T cells expanded in the presence of pVC also showed slightly reduced phosphorylation of p38 upon activation with UCHT1 as analyzed by Phosflow (**Figure 6A**, upper right). However, pVC pre-incubation increased the steady state phosphorylation of p38, which was most evident after 24 h pre-incubation [pVC (24h pre)] in Western blot and Phosflow (**Figures 6A, B**).

### 3.6 Cell surface expression of checkpoint molecules

**Figure 7:**
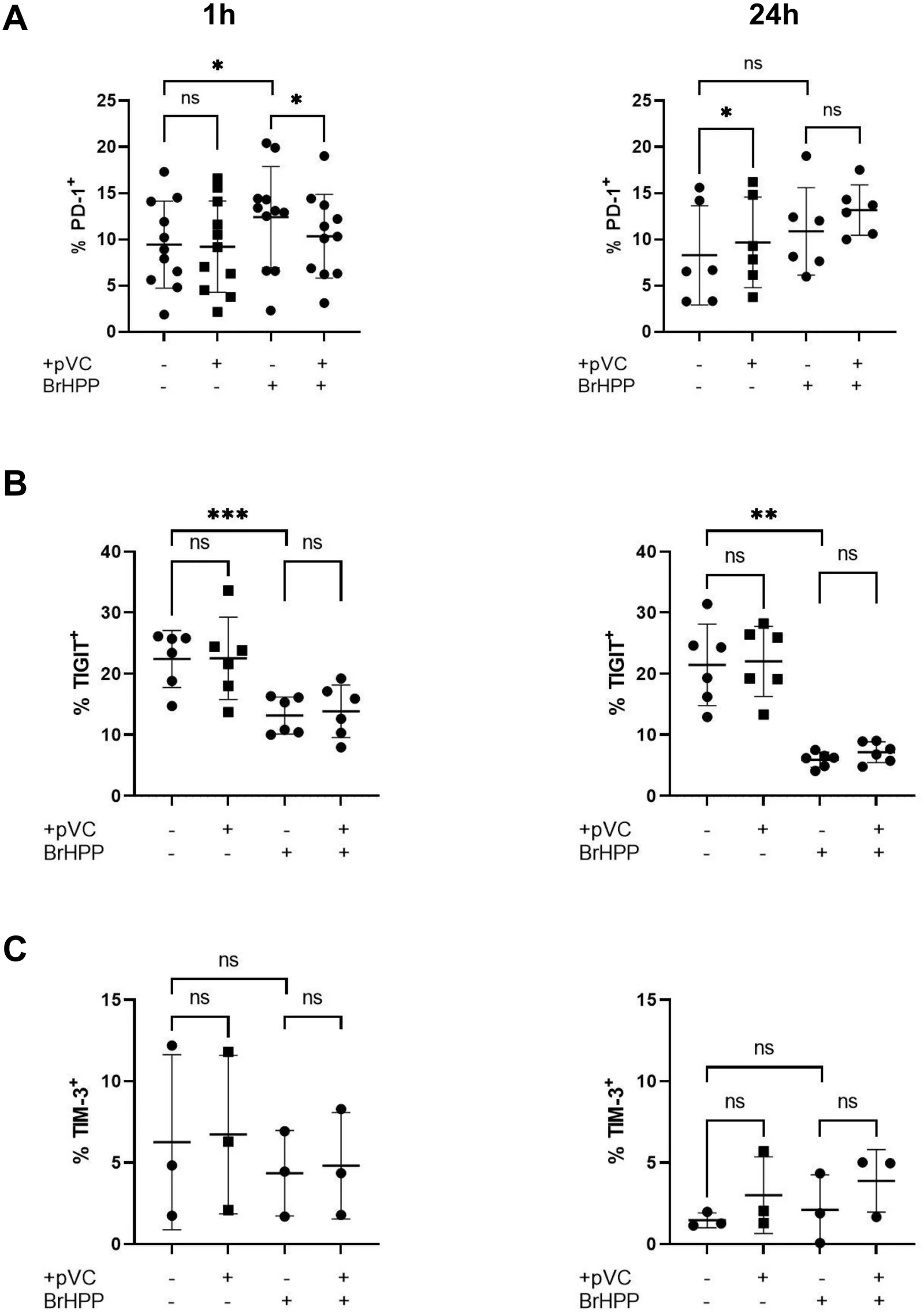
Effect of pVC on cell surface checkpoint receptor expression. Zol-expanded γδ T cells were incubated for 1 h or 24 h with 300 nM BrHPP and/or 50 μg/mL pVC as indicated. The surface expression of **(A)** PD-1, **(B)** TIGIT and **(C)** TIM-3 was analyzed by flow cytometry. Results of 6 to 11 **(A, B)** or 3 experiments **(C)**. Statistical analysis was performed by paired Student’s t test. *p<0.05, **p<0.01, ***p<0.001.

The *in vivo* efficacy of γδ T-cell based cancer immunotherapy most likely depends on critical interactions of activating and inhibitory receptors with ligands expressed in the tumor microenvironment (41). It is thus of considerable interest to investigate possible effects of pVC on the expression of checkpoint receptors on γδ T cells. To address this issue, we incubated Zol-expanded γδ T cells for 1 and 24 h in the absence or presence of pVC and TCR agonist BrHPP and analyzed the expression of PD-1, TIGIT and TIM-3. Activation with BrHPP slightly increased PD-1 expression at both time points, but this was not modulated by pVC (**Figure 7A**). TIGIT was clearly down-regulated upon BrHPP activation, but again pVC did not further modulate TIGIT expression, both in the absence and presence of BrHPP (**Figure 7B**). The expression of TIM-3 was generally low, and again we did not observe modulation by VitC (**Figure 7C**).

## 4 Discussion

TCR signaling is a complex process which is initiated upon TCR-mediated antigen recognition and subsequent activation of protein tyrosine kinases including Lck, Fyn and ZAP-70. This proximal signaling is followed by distal signaling cascades including the Ca^2+^-calcineurin-NFAT, IKK-NFκB and RAS-ERK1/2 pathways (42). Mitogen-activated protein kinases (MAPK) including ERK1/2 and p38 play important role in T-cell activation and differentiation (42). While the overall structure of the γδ TCR is similar to the αβ TCR, there are functional differences for instance with respect to the conformational change of the CD3 subunits in response to antigen challenge (30, 43). This led us to investigate the modulation of selected signaling pathways in human γδ T cells by pVC *in vitro*. The starting point of our study was the analysis of pVC effects on γδ T-cell dependent tumor cell killing in various experimental systems. In a systematic approach, we confirmed that pVC enhances the cytotoxic activity of Vγ9Vδ2 T cells against various tumor targets including pancreatic adenocarcinoma and CD19-expressing lymphoma cells. We had previously shown that pVC increases the survival of lentiviral transduced γδ T cells with anti-CD19 εTRuC (31). Our present experiments extend the previous results by demonstrating that pVC also enhances the killing capacity of γδ T cells expressing the anti-CD19 εTRuC and the secretion of IFN-γ in the lymphoma-εTRuC γδ T-cell co-cultures. This was also true when analysing anti-CD19 BBζ-CAR-expressing Vγ9Vδ2 T cells. These results support the general view that pVC enhances cytotoxic T-cell function which has been recently confirmed also in murine *in vivo* studies (44). Further, our data extend the observation that pVC treatment enhances the cytotoxicity of CAR αβ T cell cultures (32) to γδ T cells.

The activation of T cells via the TCR results in Ca^2+^ signals that are shaped by the opening of Ca^2+^ channels in the plasma membrane (45). Our results indicate that pVC can enhance the Ca^2+^ influx in expanded γδ T cells in response to TCR signaling induced by CD3 crosslinking. This effect required the pre-incubation of γδ T cells for 4 or 24 h, whereas the direct addition of VitC together with the anti-CD3 antibody resulted in variable effects, suggesting that functional modulation of Ca^2+^ channels required some time. In a previous study, it was reported that VC actually decreases intracellular calcium levels in human lymphoid cells (46). However, this study made use of a T-cell lymphoma line (Molt-3) and the calcium ionophore A23187 rather than TCR activation, and the results cannot be directly compared. Next, we analyzed the impact of pVC on the nuclear translocation of NFAT and NF-κB. Applying Imagestream cytometry on Zol-expanded γδ T cells pre-incubated or not with pVC we observed that addition of pVC alone already slightly but significantly increased the nuclear translocation of NFAT and NF-κB at the analyzed time point (4 h). While this observation was unexpected, it has been previously reported that VC upregulates NFAT in T cells (5). Moreover, VC initiates extensive demethylation at NF-kB/p65 binding sites in human dendritic cells (47), and this might also happen in γδ T cells associated with nuclear translocation of NF-κB. Overall, the precise molecular pathways modulate Ca2^+^ influx and nuclear translocation of NFAT and NF-κB requires further analysis which is beyond the goals of our current study. It remains to be investigated how VC may modulate tonic signaling, resting calcium homeostasis, redox-sensitive pathways, or epigenetic accessibility of NFAT/NF-κB target genes.

Phosphorylation of signaling molecules is a key event in early steps of T-cell activation (42). Therefore, we combined Phosflow and Western blotting to analyze the potential modulation of TCR-induced phosphorylation of CD3ζ, Akt, p38 and ERK1/2 in Vγ9Vδ2 T cells by pVC. Interestingly, phosphorylation of CD3ζ was not significantly modulated by pVC. In contrast, TCR-triggered ERK1/2 phosphorylation was reduced by pVC, particularly in γδ T-cell lines expanded in the presence of pVC. The inhibition of ERK1/2 phosphorylation revealed by both Phosflow and Western blotting is in line with the reported inhibition of ERK1/2 activation by VC in other cells such as myoblasts and thyroid cancer cells (48, 49). In addition to VC, the elevated steady state p38 phosphorylation in pVC treated Vγ9Vδ2 T cells might add to the reduced ERK1/2 phosphorylation, since both pathways are known to be interlinked, and p38 was shown to inhibit ERK phosphorylation in non-immune cells (cardiac myocytes) (50). Interestingly, ERK signaling has been shown to antagonize Ca2+ influx in human T cells upon TCR stimulation (51, 52). Therefore, the VC-dependent reduction of ERK phosphorylation could contribute to the pronounced Ca2^+^ influx observed in this study. ERK phosphorylation is known to be a promoting factor of T-cell proliferation and effector functions such as IFN-γ production (53). However, it also has been demonstrated that ERK-dependent signaling in strongly stimulated T cells leads to the suppression of proliferation (54), the VC induced reduced ERK phosphorylation observed by us, could therefore mechanistically explain the proliferation promoting effect by VC reported by us before for Vδ2 T cells (19) and for CAR αβ T cells by Rahmati et al. (32).

Finally, we have analyzed the potential effect of pVC on the expression of checkpoint receptors on *in vitro* expanded γδ T cells. Inhibitory receptors, are important parameters to be considered in the context of application of γδ T cells in cancer immunotherapy (41). We compared the expression of PD-1, TIGIT and TIM-3 on Zol-expanded γδ T cells incubated for 1 or 24 h with pVC in the absence or presence of the TCR agonist BrHPP. These experiments revealed a clear down-modulation of TIGIT expression in response to BrHPP but no modulation by pVC. Similarly, the variable expression of PD-1 and TIM-3 was not influenced by pVC. These observations have important implications for the use of pVC to boost γδ T-cell functionality for the purpose of adoptive transfer into cancer patients. Our results confirm that pVC does not induce upregulation of such checkpoint receptors which otherwise might interfere with the efficacy of transferred γδ T cells *in vivo*.

Our study is the first to address the effect of pVC on selected signaling pathways in human γδ T cells. Increased Ca^2+^ influx, nuclear translocation of NFAT and NF-κB, and modulation of kinase activation might all contribute to the enhanced functionality of γδ T cells in response to pVC. Further studies are required to better understand the molecular mechanisms how pVC enhances T-cell function including the effector function of γδ T cells. In this respect, it will be important to investigate the impact of VC and pVC on signaling pathways in γδ T cells *in vivo* in tumor models in humanized mice. Since high-dose i.v. dosage of VC is applied in clinical studies (55, 56), this also offers the opportunity to address the relevance of our findings under ex vivo conditions. In addition to its activity as anti-oxidant and epigenetic modifier, recent data suggest that VC also induces post-translational modifications of signaling proteins which might be another contributing factor to boosting immunity (57). Understanding in greater detail the molecular mechanisms of pVC and VC will help to broaden its application in cancer immunotherapy beyond the γδ T-cell perspective (11, 13, 58–60).

## Data Availability

The raw data supporting the conclusions of this article will be made available by the authors, without undue reservation.
