# Supplementary material for: Vitamin C enhances cytotoxicity of CAR-and εTRuC-engineered γδ T cells and modulates Ca2+, NFAT, NF-κB and ERK signaling in human γδ T cells
Source: Front Immunol. 2026 Aug 28;17:1759941. doi: 10.3389/fimmu.2026.1759941 (PMC13561771; doi:10.3389/fimmu.2026.1759941)
Supplement: Supplementary file 1 [file DataSheet1.pdf]

Supplementary Materials for

Vitamin C enhances cytotoxicity of CAR- and εTRuC-engineered γδ T cells and modulates Ca^{2+}, NFAT, NF-κB and ERK signaling in human γδ T cells

Michal Zarobkiewicz, Lin Ma, Claudia Juraske, Lea Cierna *et al.*

Content: page

Figure S1. Phenotypic characterization of Vγ9Vδ2 T cells expanded in the absence or presence of pVC. 2

Figure S2. Flow cytometric characterization and donor-specific cytotoxic activity of mock- and CAR-transduced Vγ9Vδ2 T cells. 3

Figure S3. Flow cytometric characterization and donor-specific cytotoxic activity of mock- and γδ εTRuC-transduced Vγ9Vδ2 T cells. 4

Figure S4. Effect of pVC on NFAT- and NF-κB nuclear translocation 24 h after restimulation in Vγ9Vδ2 T cells. 5

Figure S5. Semi-quantitative analysis of ERK1/2 phosphorylation by Western blot densitometry. 7

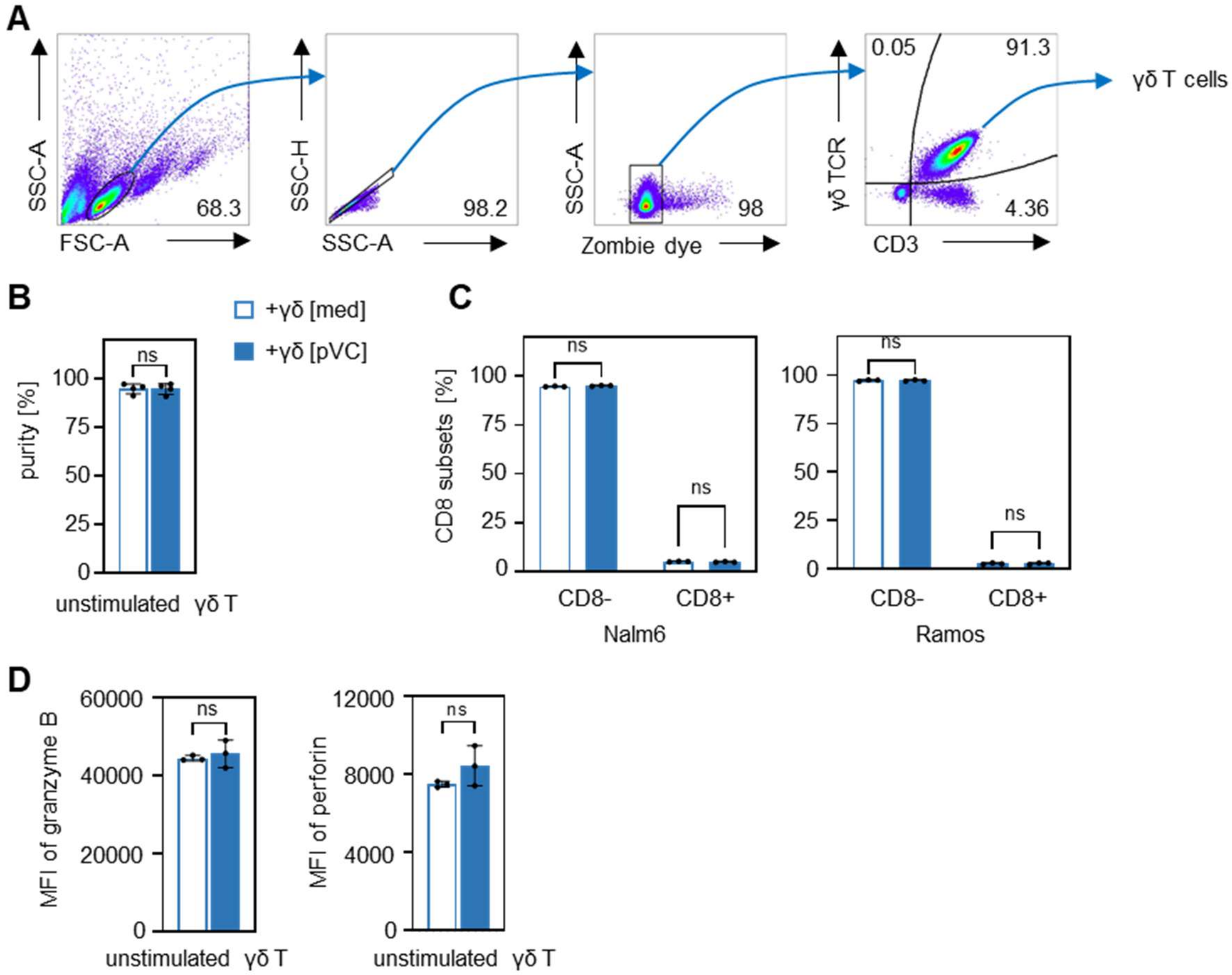

Figure S1. Phenotypic characterization of Vγ9Vδ2 T cells expanded in the absence or presence of pVC.

(A) Flow cytometry gating for our Zol-expanded Vγ9Vδ2 T cells. Cells were first gated on forward scater/side scatter (FSC/SSC) to exclude debris, followed by singlet gating and live/dead discrimination (Zombie dye). γδ T cells were defined as $CD3^+$ $γδTCR^+$. Percentages indicate the fraction of cells within the respective parent gate. **(B)** Purity of the γδ T cells after 14 days of expansion with Zol in the absence or presence of pVC. The purity of Vγ9Vδ2 T cells was around 85-95% and is similar with (γδ [pVC]) and without pVC (γδ [med]). Purity was defined as the percentage of $CD3^+$ $γδTCR^+$ cells in the right panel of (A). Each dot represents one independent donor, and bars indicate the mean ± SD. Statistical significance was assessed using a paired t test (n = 4); ns, not significant. **(C)** Frequencies of $CD8^-$ and $CD8^+$ Vγ9Vδ2 T cells followed by a 24 h of co-culture with Nalm6 or Ramos cells. Statistical significance was assessed using two-way ANOVA (n = 3; one representative experiment performed in triplicate is shown). ns, not significant. **(D)** Intracellular granzyme B and perforin expression in Vγ9Vδ2 T cells cells, quantified as median fluorescence intensity (MFI). Statistical significance was assessed using an unpaired t test (n = 3; one representative experiment performed in triplicate is shown). ns,not significant.

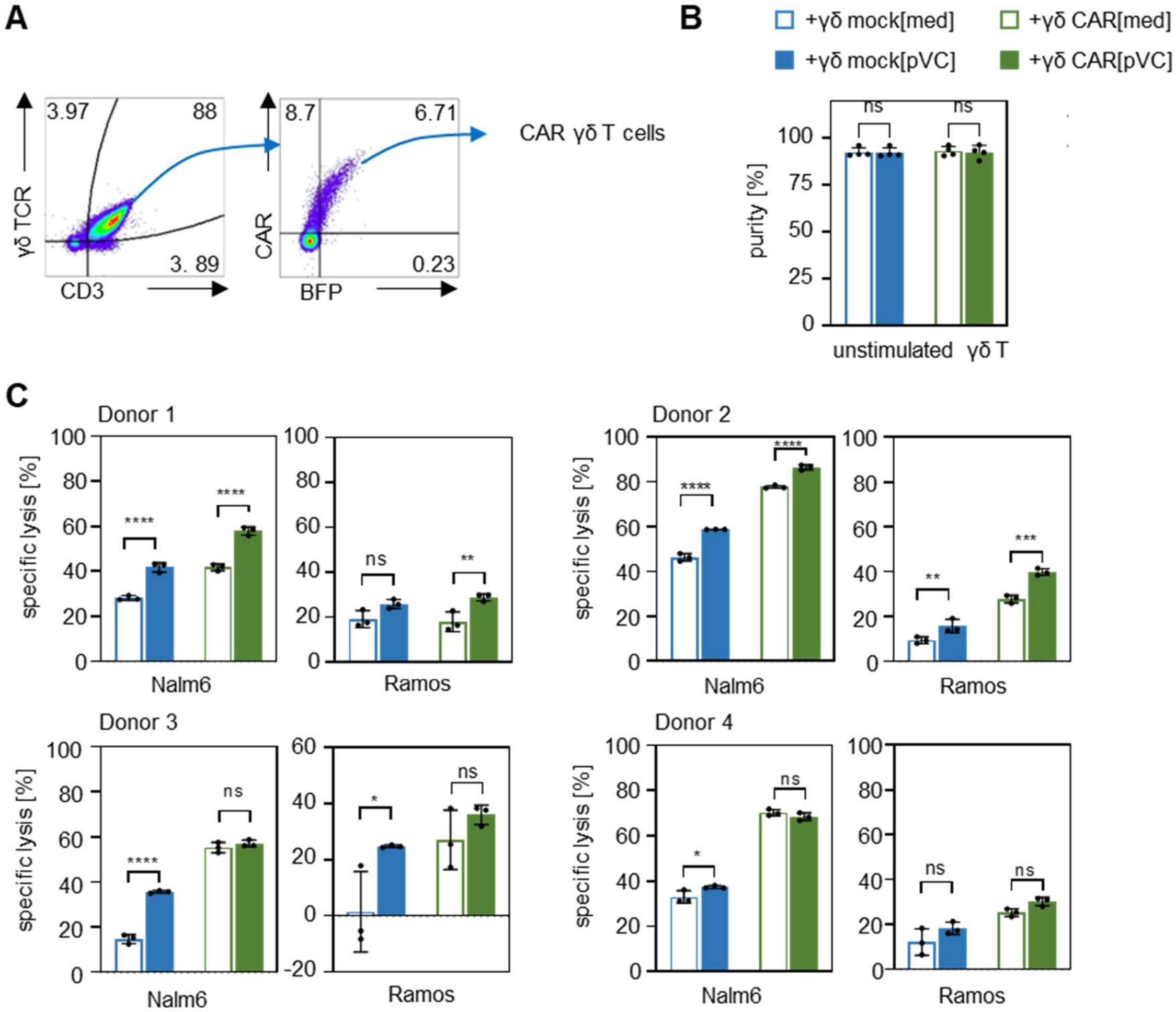

Figure S2. Flow cytometric characterization and donor-specific cytotoxic activity of mock- and CAR-transduced Vγ9Vδ2 T cells.

(A) Flow cytometry gating strategy for γδ CAR T cells. After gating on life cells as in figure S1A, Vγ9Vδ2 T cells were defined as $CD3^{+}$ $\gamma\delta TCR^{+}$, Transduction efficiency was assessed by BFP and CAR expression by an anti-flag tag stain (CAR). **(B)** Purity of mock- and CAR-transduced Vγ9Vδ2 T cells after 14 days of Zol-expansion in medium alone or in the presence of pVC. Purity was defined as in figure S1B and was around 85-95%. Statistical significance was assessed using a paired t test (n = 4. **(C)** Specific lysis of Nalm6 and Ramos target cells mediated by mock- or CAR-transduced Vγ9Vδ2 T cells Zol-expanded for 14 days in medium alone or in the presence of pVC. Cytotoxicity was assessed after 8 h of co-culture at an effector-to-target (E:T) ratio of 1:1 using a luciferase-based cytotoxicity assay. Results from four independent donors are shown separately. Each dot represents one technical replicate, and bars indicate the mean ± SD of triplicate measurements. Statistical significance was assessed separately for each donor using two-way ANOVA. ** $p<0.01$, *** $p<0.001$, **** $p<0.0001$.

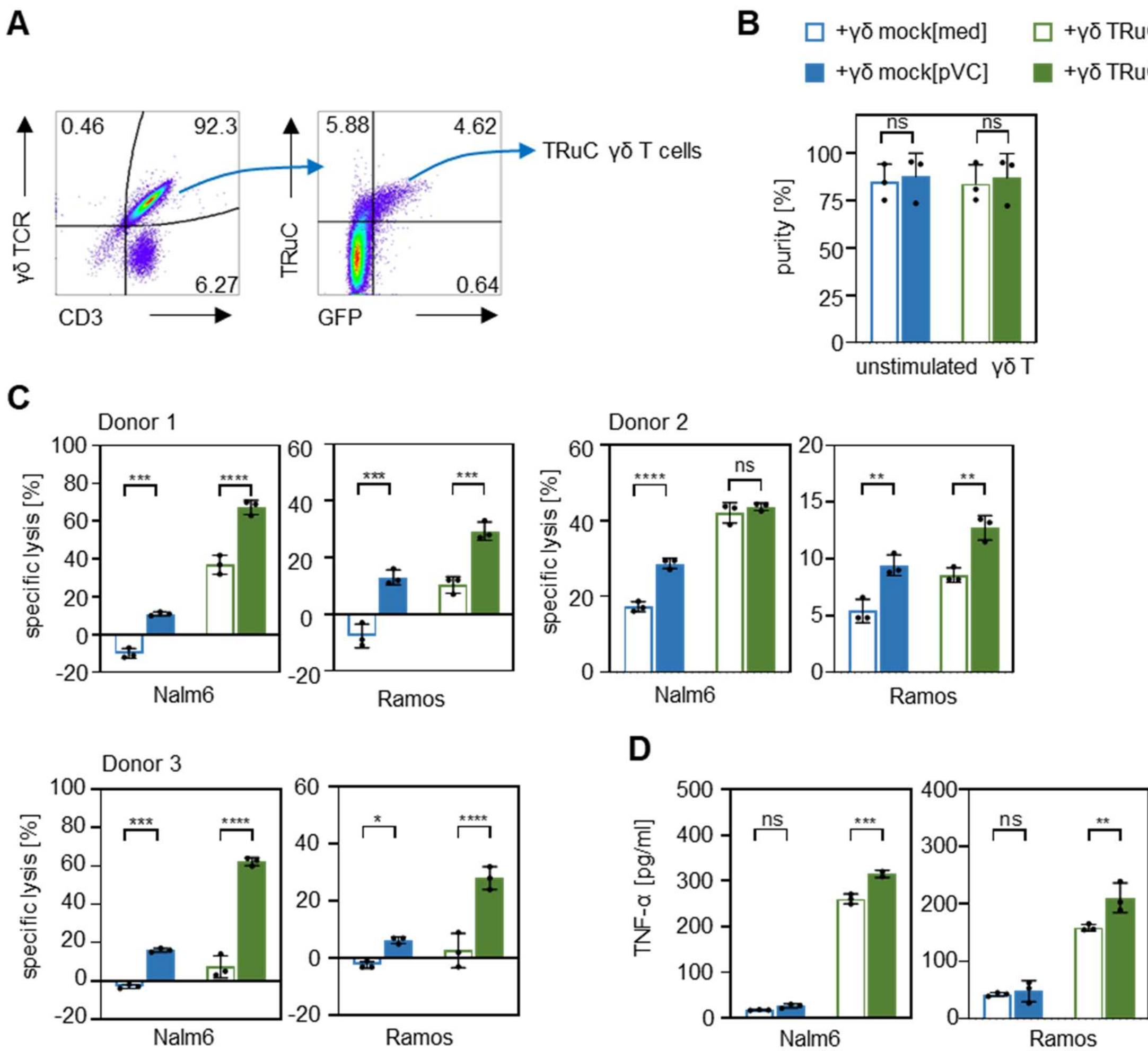

Figure S3. **Flow cytometric characterization and donor-specific cytotoxic activity of mock- and γδ ε TRuC-transduced Vγ9Vδ2 T cells.**

(A) Flow cytometry gating strategy for γδ εTRuC T cells. After gating on life cells as in figure S1A, Vγ9Vδ2 T cells were defined as $CD3^{+}$ $\gamma\delta TCR^{+}$, Transduction efficiency was assessed by GFP and εTRuC expression by an anti IgG F(ab') 2 stain (εTRuC). **(B)** Purity of mock- and εTRuC-transduced Vγ9Vδ2 T cells after 14 days of Zol-expansion in medium alone or in the presence of pVC. Purity was defined as in figure S1B and was around 85-95%. Statistical significance was assessed using paired t test (n =3). **(C)** Specific lysis of Nalm6 and Ramos target cells mediated by mock- or εTRuC-transduced Vγ9Vδ2 T cells Zol-expanded in medium alone or in the presence of pVC. Cytotoxicity was assessed after 8 h of co-culture at an effector-to-target (E:T) ratio of 1:1 using a luciferase-based cytotoxicity assay. Results from three independent donors are shown separately. **(D)** After 24 h, the supernatants of the co-cultures were used to determine TNF-α. The significance was calculated by two-way ANOVA (n=3; one representative experiment in triplicates is shown). * $p<0.05$, ** $p<0.01$, *** $p<0.001$, **** $p<0.0001$.

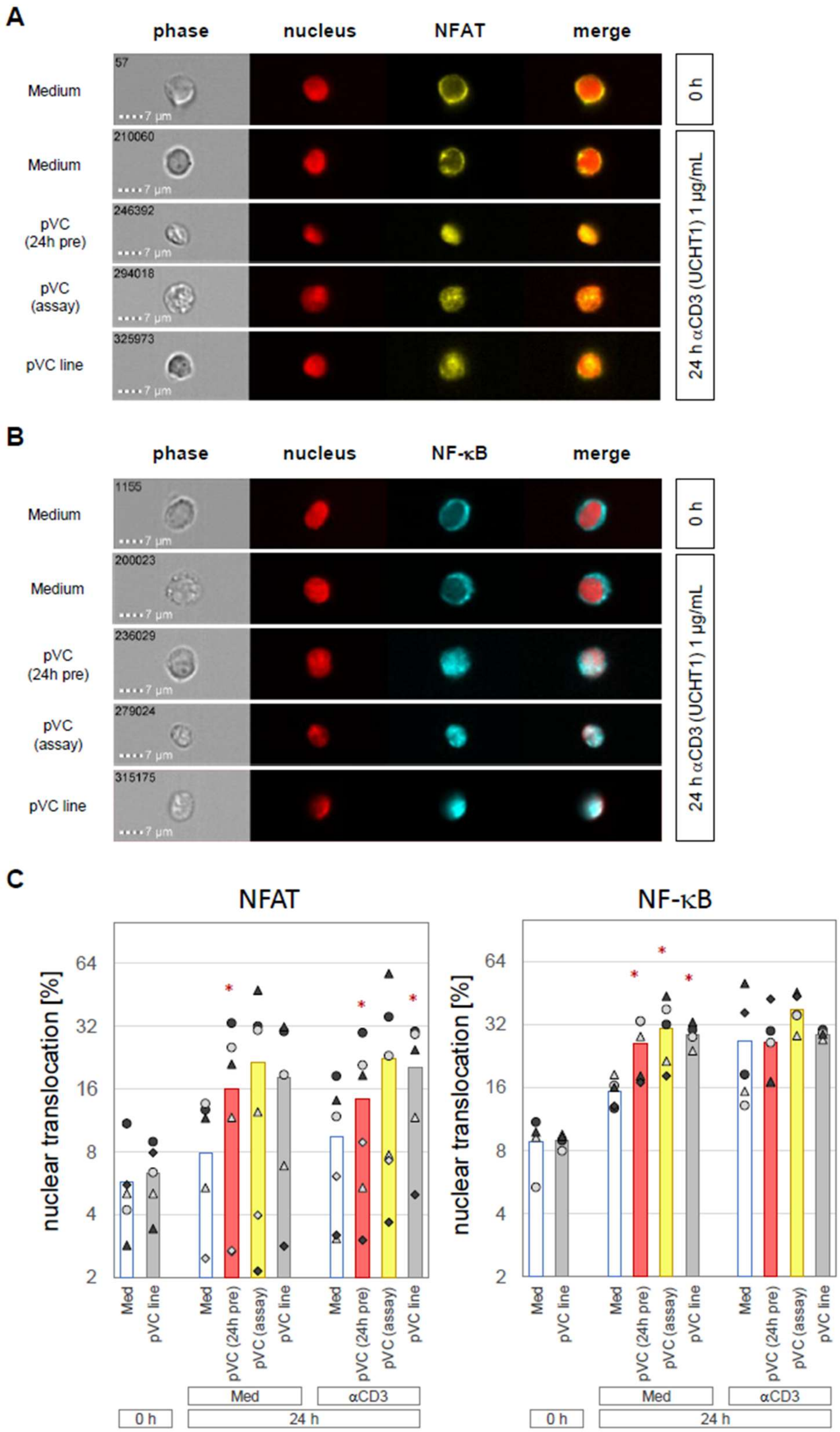
A
phase
nucleus
NFAT
merge
Medium
Medium
pVC
(24h pre)
pVC
(assay)
pVC line
0 h
24 h αCD3 (UCHT1) 1 µg/mL
B
phase
nucleus
NF-κB
merge
Medium
Medium
pVC
(24h pre)
pVC
(assay)
pVC line
0 h
24 h αCD3 (UCHT1) 1 µg/mL
C
NFAT
NF-κB
nuclear translocation [%]
64
32
16
8
4
2
Med
pVC line
pVC (24h pre)
pVC (assay)
Med
αCD3
0 h
24 h

Figure S4. Effect of pVC on NFAT- and NF-κB nuclear translocation 24 h after restimulation in Vγ9Vδ2 T cells.

Vγ9Vδ2 γδ T cells were expanded with Zol in the absence [Medium] or presence of pVC [pVC line]. After the expansion, γδ T cells were pre-treated or not with pVC for 24 h [pVC (24h pre)]. The γδ T cells were then re-stimulated for 24 h with anti-CD3 antibody UCHT1. To some of the [Medium] γδ T cells, pVC was added during this re-stimulation [pVC assay]. **(A** and **B)** The translocation of NFAT (yellow) and NF-κB (blue) into the nucleus (red) was analyzed using Image Stream flow cytometric microscopy. A representative picture of the nuclear translocation of NFAT (A) or NF-κB (B) after 0 or 24 h is shown for all different pVC treatments [Medium, pVC (24 h pre), pVC assay, pVC line]. **(C)** The percentages of nuclear translocation after 0 and 24 h are shown for NFAT (n=6) and NF-κB (n=5). Columns represent mean values; asterisks indicate significance levels; * $p \leq 0.05$, ** $p \leq 0,01$.

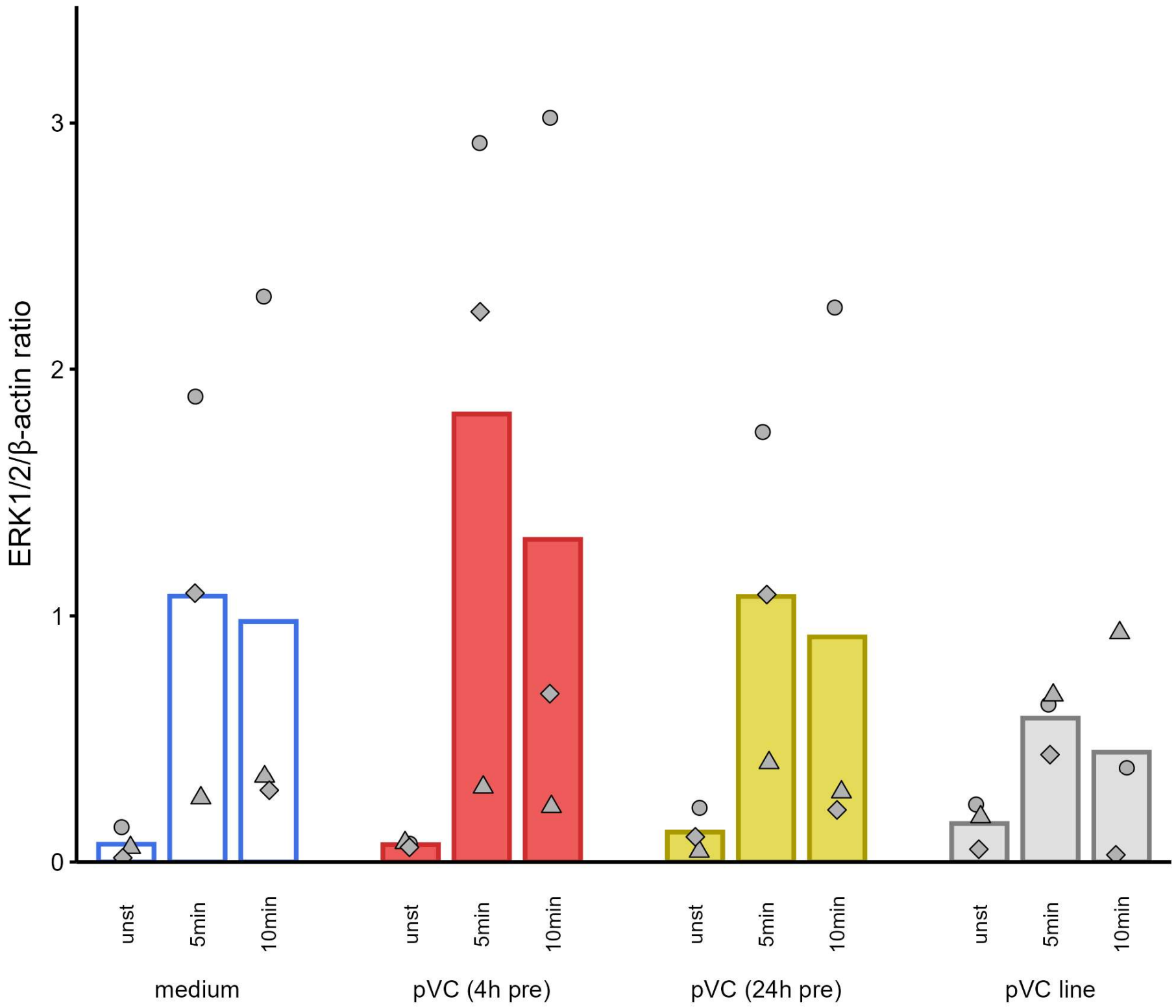

Figure S5. Semi-quantitative analysis of ERK1/2 phosphorylation by Western blot densitometry. Phosphorylated ERK1/2 abundance was quantified in samples of ZOL-expanded γδ T cells pre-cultured or not with 50 µg/mL pVC for 4 or 24 h as indicated, or expanded in the presence of pVC. The cells were left untreated or were stimulated for 5 min or 10 min with anti-CD3 antibody UCHT1. Densitometric values were normalized to β-actin and are presented as ERK1/2/β-actin ratios. Bars show mean values, while individual symbols represent three independent biological samples.
